# Automatic colorimetric calibration of human wounds

**DOI:** 10.1186/1471-2342-10-7

**Published:** 2010-03-18

**Authors:** Sven Van Poucke, Yves Vander Haeghen, Kris Vissers, Theo Meert, Philippe Jorens

**Affiliations:** 1 Department of Anaesthesia, Critical Care, Emergency Care, Genk, Belgium; 2 Department of Dermatology, University Ghent, Ghent, Belgium; 3 Department of Anesthesiology, Pain and Palliative Medicine, The Radboud University Nijmegen Medical Centre, Nijmegen, Netherlands; 4 CNS, Pain and Neurology, Janssen Research Foundation, Beerse, Belgium; 5 Critical Care Department, Antwerp University Hospital, University of Antwerp, Antwerp, Belgium

## Abstract

**Background:**

Recently, digital photography in medicine is considered an acceptable tool in many clinical domains, e.g. wound care. Although ever higher resolutions are available, reproducibility is still poor and visual comparison of images remains difficult. This is even more the case for measurements performed on such images (colour, area, etc.). This problem is often neglected and images are freely compared and exchanged without further thought.

**Methods:**

The first experiment checked whether camera settings or lighting conditions could negatively affect the quality of colorimetric calibration. Digital images plus a calibration chart were exposed to a variety of conditions. Precision and accuracy of colours after calibration were quantitatively assessed with a probability distribution for perceptual colour differences (dE_ab). The second experiment was designed to assess the impact of the automatic calibration procedure (i.e. chart detection) on real-world measurements. 40 Different images of real wounds were acquired and a region of interest was selected in each image. 3 Rotated versions of each image were automatically calibrated and colour differences were calculated.

**Results:**

1^st ^Experiment: Colour differences between the measurements and real spectrophotometric measurements reveal median dE_ab values respectively 6.40 for the proper patches of calibrated normal images and 17.75 for uncalibrated images demonstrating an important improvement in accuracy after calibration. The reproducibility, visualized by the probability distribution of the dE_ab errors between 2 measurements of the patches of the images has a median of 3.43 dE* for all calibrated images, 23.26 dE_ab for all uncalibrated images. If we restrict ourselves to the proper patches of normal calibrated images the median is only 2.58 dE_ab! Wilcoxon sum-rank testing (p < 0.05) between uncalibrated normal images and calibrated normal images with proper squares were equal to 0 demonstrating a highly significant improvement of reproducibility. In the second experiment, the reproducibility of the chart detection during automatic calibration is presented using a probability distribution of dE_ab errors between 2 measurements of the same ROI.

**Conclusion:**

The investigators proposed an automatic colour calibration algorithm that ensures reproducible colour content of digital images. Evidence was provided that images taken with commercially available digital cameras can be calibrated independently of any camera settings and illumination features.

## Background

Chronic wounds are a major health problem, not only because of their incidence, but also because of their time- and resource-consuming management. This study was undertaken to investigate the possible use of colorimetric imaging during the assessment of human wound repair. The outline design of the current study is based on the system requirements for colorimetric diagnostic tools, published previously [1,2].

**Figure 1:**
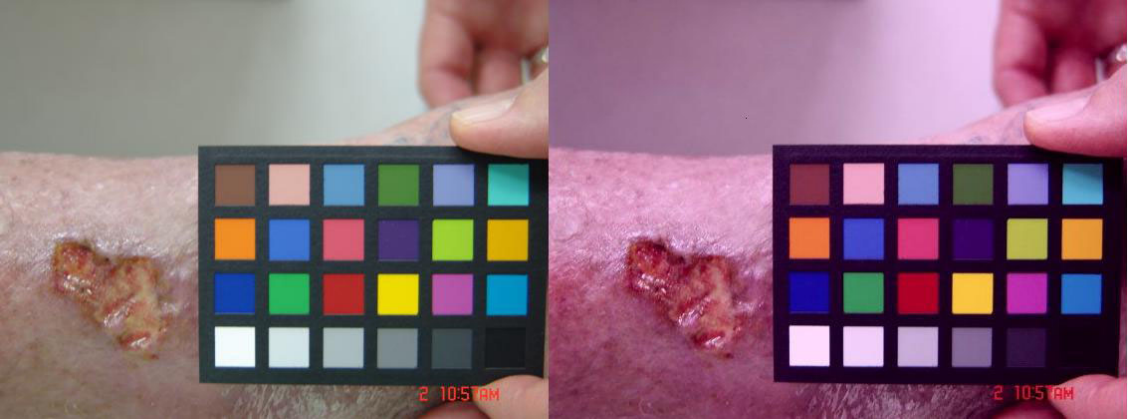
**Chronic Wound and Reference Chart**. Chronic Wound and Reference Chart after (left) and before (right) calibration.

Digital photography is considered an acceptable and affordable tool in many clinical disciplines such as wound care and dermatology [3-12], forensics [13,14], pathology [15], traumatology, and orthodontics [16,17]. Although the technical features of most digital cameras are impressive, they are unable to produce reproducible and accurate images with regard to spectrophotometry [18-22]. Taking two pictures of a wound with the same camera and settings, immediately after one another, normally results in two slightly different images. These differences are exacerbated when the lighting, the camera or its settings are different. Therefore, reproducibility is poor. This may be less important when photographs are taken for documentation purposes, but when digital photography becomes part of medical evaluation or is used to perform measurements, it becomes critically important [5,6,18,23-29]. In our view, the quality of medical photography is principally defined by its reproducibility and accuracy [21]. Without reproducibility and accuracy of images, any attempt to measure colour or geometric properties is of little use [27]. A simple, practical and validated algorithm to solve this problem is necessary (Figure 1).

Almost all colours can be reconstructed using a combination of three base colours; red, green and blue (RGB) [30]. Together, these three base colours define a 3-dimensional colour space that can be used to describe colours.

The accurate handling of colour characteristics of digital images is a non-trivial task because RGB signals generated by digital cameras are 'device-dependent', i.e. different cameras produce different RGB signals for the same scene. In addition, these signals will change over time as they are dependent on the camera settings and some of these may be scene dependent, such as the shutter speed and aperture diameter. In other words, each camera defines a custom device-dependent RGB colour space for each picture taken. As a consequence, the term RGB (as in RGB-image) is clearly ill-defined and meaningless for anything other than trivial purposes. As measurements of colours and colour differences in this paper are based on a standard colorimetric observer as defined by the CIE (Commission Internationale de l'Eclairage), the international standardizing body in the field of colour science, it is not possible to make such measurements on RGB images if the relationship between the varying camera RGB colour spaces and the colorimetric colour spaces (colour spaces based on said human observer) is not determined. However, there is a standard RGB colour space (sRGB) that is fixed (device-independent) and has a known relationship with the CIE colorimetric colour spaces. Furthermore, sRGB should more or less display realistically on most modern display devices without extra manipulation or calibration (look for a 'sRGB' or '6500K' setting) [31]. One disadvantage of sRGB is that it cannot represent all the colours detected by the human eye. We believe that finding the relationship between the varying and unknown camera RGB and the sRGB colour space will eliminate most of the variability introduced by the camera and lighting conditions.

**Figure 2:**
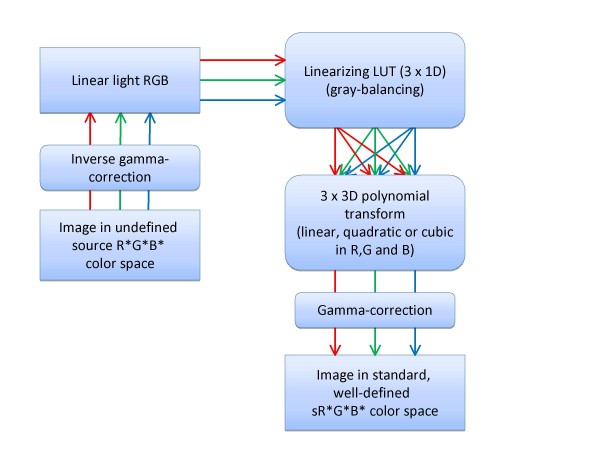
**RGB to sRGB Transformation Scheme**.

The transformation between the input RGB colour space and the sRGB colour space was achieved via a colour target-based calibration using a 'reference chart', namely the MacBeth Colour Checker Chart Mini [MBCCC] (GretagMacBeth AG, Regensdorf, Switzerland). This chart provides a checkerboard array of 24 scientifically prepared coloured squares or patches in a wide range of colours with known colorimetric properties under a CIE D65, noon daylight illuminant (6504 K). Many of these squares represent natural objects of special interest, such as human skin, foliage and blue sky. These squares are not only the same colour as their counterparts, but also reflect light the same way in all parts of the visible spectrum. Different calibration algorithms defining the relationship between the input RGB colour space of the camera and the sRGB colour space have been published using various methods such as 3D look-up tables and neural networks. The algorithm in this study is based on three 1D look-up tables and polynomial modelling, as previously published by Vander Haeghen et al. [32] (Figure 2). This is a little different than e.g. the general methods used in the well-known ICC profiles http://www.color.org/index.xalter. In ICC profiles the relationship of an unknown colour spaces to the so-called 'profile connection space' (PCS, usually CIE XYZ) are computed and stored. Output is then generated by going from this PCS to the desired output colour space, which in our case would be sRGB. This means 2 colour space transformations are required (RGB to PCS to sRGB), while our algorithm only needs 1. Although an inherently more flexible system, ICC profiling seems overkill for our intended application (straight camera RGB to sRGB transformation, without the need of determining and storing or embedding a device profile). However, It must be said that the advent of e.g. LittleCMS http://www.littlecms.com/ which is a free colour management system that focuses on determination and immediate application of profiles on images may change this view in the future, and that such a system could be a viable alternative for the current colour space transformation algorithms in our system.

## Methods

The research has been carried out in accordance with the Helsinki Declaration; the methods used were subject to ethical committee approval (B32220083450 Commissie voor Medische Ethiek Faculteit Geneeskunde Leuven Belgium). Patients received detailed written and verbal explanation and patient authorization was required before inclusion and analysis of the images.

### Experiment 1

The purpose of the first experiment was to investigate whether camera settings or lighting conditions negatively affect the quality of the colorimetric calibration [33]. Chronic wounds are assessed in different locations and environments. Therefore, we assessed the calibration algorithm under extreme lighting conditions and with inappropriate camera settings.

#### Image Acquisition

Digital images of the MBCCC on a grey-coloured background, in a Colour Assessment Cabinet CAC 120-5 (VeriVide Leicester, UK), were taken using two digital cameras; the Nikon D200 SLR (10.2 effective mega pixels) with a 60 mm AF Micro Nikkor lens, and the Canon Eos D10 (6.3 million effective pixels) with a 50 mm Canon EF lens. All images were processed in high-quality jpeg mode. This means after the camera has applied processing (demosaicing, colour correction curves, matrixing, etc. ...) to the images. (Table 1)

**Table 1:** Parameter Settings

Camera Type	Canon Eos D10	Nikon D200		
Scene lighting (cabinet)	D65	TL84	A	
Camera sensitivity	100 ISO	400 ISO		
Camera exposure	-1EV	0EV	+1EV	
Camera white balance	Auto	Manual	A	D65

Lighting and camera settings records all the parameters that were varied during image acquisition. Note that these include some combinations that are inappropriate, such as setting the camera white balance to D65 with an A scene illuminant to produce off-colour images. This was done in order to challenge the calibration algorithm. 1^st ^Illuminant D65: 'Artificial Daylight' fluorescent lamps conforming to Standard Illuminant D6500 (6500 K), 2^nd ^Illuminant TL84: Philips Triphosphor fluorescent lamps, often chosen as a 'Point of Sale' illuminant, (5200 K), 3^rd ^Illuminant A: 'Filament (domestic) lighting' (3000 K). In digital photography, ISO measures the sensitivity of the image sensor. Exposure is measured in lux seconds, and can be computed from exposure value (EV) and scene luminance over a specified area. White balancing is a feature that allows to compensate for different lighting conditions by adjusting the color balance based on the difference between a white object in the image and a reference white.

#### Calibration Procedure

During the calibration procedure uniform illumination is assumed, as is a reference chart as part of the image of interest. The calibration provides a means of transforming the acquired images (defined in an unknown colour space, which is normally RGB), to a standard, well-defined colour space i.e. sRGB [34]. sRGB has a known relationship to the CIE L*a*b* colorimetric space, allowing computation of perceptual colour differences. The CIE L*a*b* colorimetric space, or CIELAB space with coordinates L*, a* and b*, refers to the colour-opponent space; L* refers to Luminance, a* and b* refer to the colour-opponent dimensions [34-36]. The 'detection of the MBCCC' in the digital image can be done manually or automatically. The algorithm behind MBCCC detection is based on the initial detection of all the bright areas in an image (areas with pixel values close to 255), followed by a shape analysis. Shapes that are not rectangular, and either too small or too large compared with the image dimensions, are discarded (in pixel, we do not know the real dimension yet). The remaining areas are candidates for the MBCCC white patch. For each of the white patch candidates, the corresponding MBCCC black patch is searched for, taking into account the typical layout of the colour chart and the dimensions of the white patch candidate. If this succeeds, the patches are checked for saturation (average pixel value > 255-δ or < \delta with \delta a small number, e.g. 3) in each of the colour channels individually. If the number of saturated patches is acceptable (typically fewer than 6 out of 24 patches), calibration proceeds and its quality is assessed. Quality assessment consists of examining various conditions relating to the colour differences between the known spectrophotometric and the computed sRGB values, in accepted and rejected patches. If any of these tests fail, the algorithm rejects the calibration and continues the search.

#### Analysis

In this experiment precision is defined as a measure of the proximity of consecutive colour measurements on an image of the same subject. This is also known as reproducibility. The precision of the MBCCC chart detection, together with the calibration process, were evaluated by computing the perceptual colour differences between all the possible pairs of measurements of each colour square of the MBCCC chart. These perceptual colour differences are expressed in CIE units, and are computed using the Euclidean metric in the CIE L*a*b* colour space. Theoretically, one unit is the 'just noticeable colour difference' and anything above five units is 'clearly noticeable'.

The accuracy of a procedure is a measure of how close its results are to the 'real' values, i.e. those obtained using the 'standard' procedure or measurement device. For colour measurements this would be a spectrophotometer. Consequently, the accuracy of the chart detection and colour calibration can be assessed by computing the perceptual colour differences between the measurements of the colour squares of the MBCCC chart and the spectrophotometric values of these squares. For this assessment the calibration was performed using half the colour patches of the MBCCC chart, while the other half were utilised in evaluation of accuracy. Accuracy is likely to be higher when the whole chart is used for calibration purposes. Precision and accuracy result in a probability distribution for the dE_ab errors. Tukey's five-number summary of the dE_ab colour differences of each patch was also calculated and visualized using a box plot (the minimum, the lower quartile, the median, the upper quartile and the maximum). Wilcoxon rank-sum statistics were used to test the calibration, which compares the locations of two populations to determine if one population has been shifted with respect to another. A sum of ranks comparison, which works by ranking the combined data sets and summing the ranks for each dE_ab, was utilised to compare the sum of the ranks with significance values based on the decision alpha (p < 0.05).

### Experiment 2

**Figure 3:**
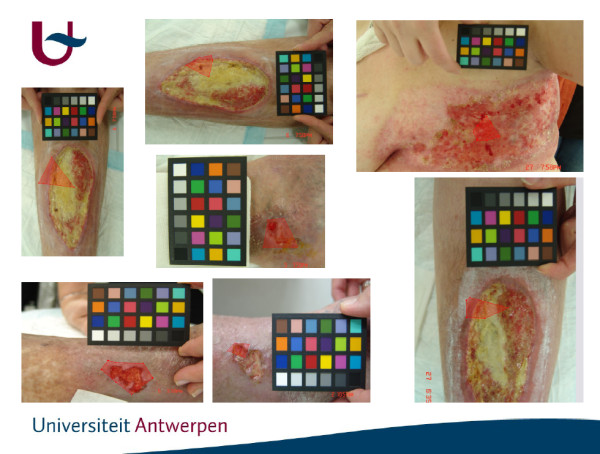
**Chronic Wound Images with Reference Chart and a Region of Interest**.

The second experiment was designed to quantify the impact of the automatic calibration procedure i.e. the chart detection, on real-world measurements. This may be of importance in a clinical setting, where automatic calibration of large batches of images in a single run is required. To examine this, 40 different images of real wounds were acquired, and a region of interest (ROI) was selected within each image. Three rotated versions (at 90°, 180° and 270°) of each image were created and automatically calibrated (Figure 3). Comparisons between the colour measurements of the ROIs of the rotated versions of each image highlighted the errors introduced by the automatic chart detection component of the calibration procedure.

#### Image Acquisition

Digital images (n = 40) of the chronic wounds were taken using a Sony Cybershot DSC-F828 digital camera (8.0 million effective pixels) and Carl Zeiss 28 - 200 mm equivalent lens, with fully automatic settings at different indoor locations, as is usually the case in daily clinical practice.

#### Calibration Procedure and Analysis

The calibration procedure was carried out in accordance with that recorded for experiment 1. The dE_ab colour differences between the average colour of the ROI of the four rotated versions of each image were computed and visualized using a probability distribution graph.

## Results

### Experiment 1

**Figure 4:**
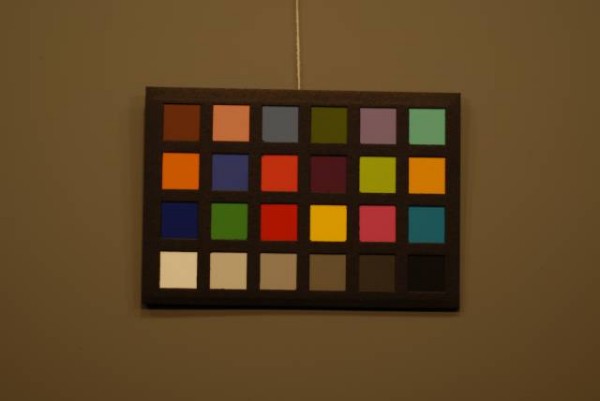
**Example with Nikon D200: MBCCC under illuminant A (3000 K)**. Camera: exposure bias -1, automatic white balance.

**Figure 5:**
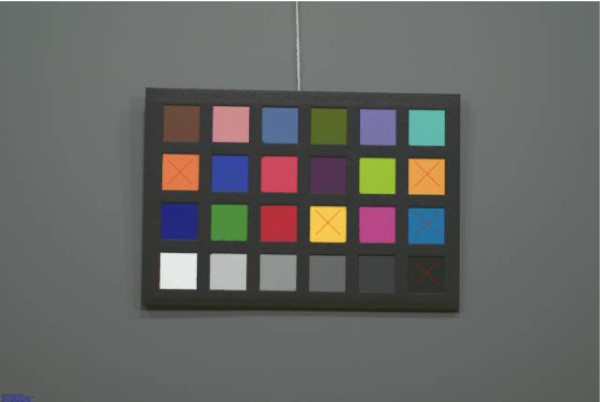
**Example with Nikon D200: MBCCC under illuminant A (3000 K)**. Camera: exposure bias -1, calibrated image.

**Figure 6:**
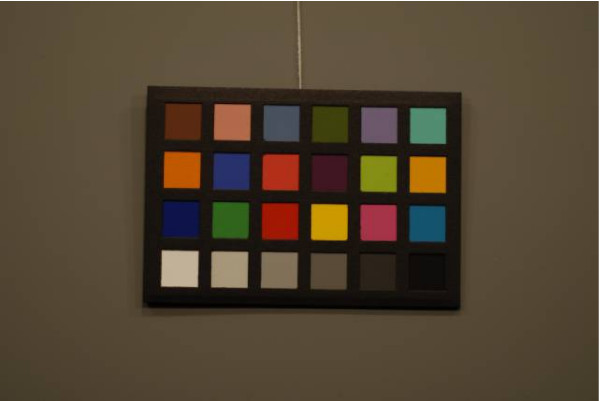
**Example with Canon 10D: MBCCC under illuminant A (3000 K)**. Camera: exposure bias -1, manual white balance.

**Figure 7:**
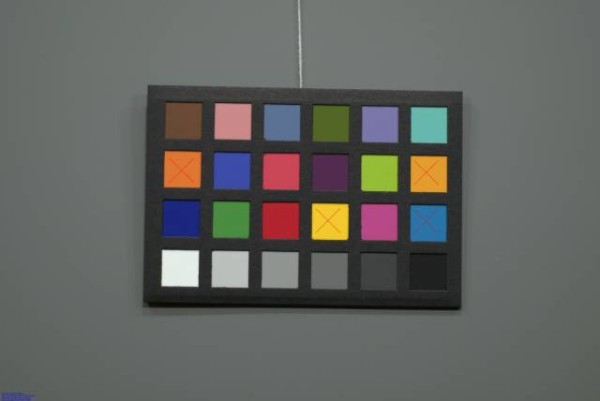
**Example with Canon 10D: MBCCC under illuminant A (3000 K)**. Camera: exposure bias -1, calibrated image.

**Figure 8:**
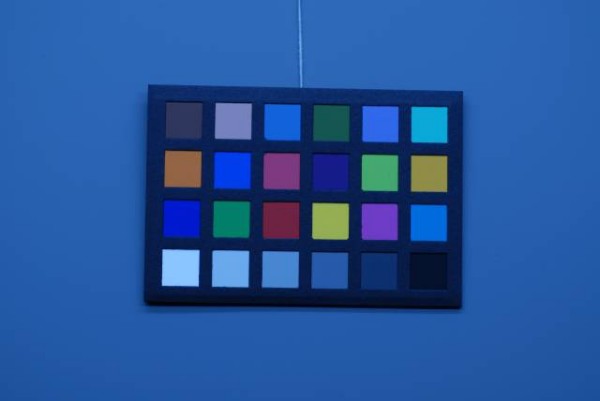
**Example with Nikon D200: MBCCC under illuminant D65 (6500 K)**. Camera: exposure bias -1, manual white balance.

**Figure 9:**
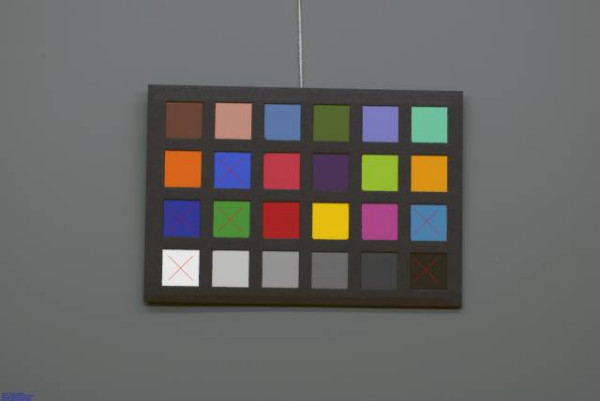
**Example with Nikon D200: MBCCC under illuminant D65 (6500 K)**. Camera: exposure bias -1, calibrated image.

Figures 4, 5, 6, 7, 8 and 9 demonstrate examples of realistic sample images under different illuminants and show the corresponding calibrated images taken with different cameras. The images contained many saturated patches (see the 'x's on the patches) that were not used for the calibration, resulting in a lower quality calibration.

**Figure 10:**
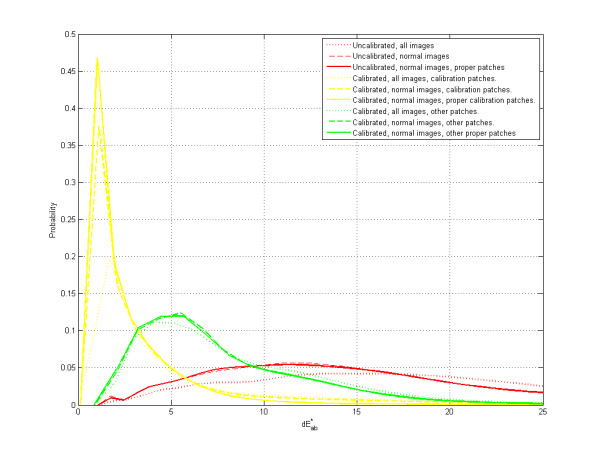
**Accuracy of Color Calibration**. Probability distribution of dE*ab errors between the patches of the images and spectrophotometric measurements. Based on 39 images (Nikon D200) & 15 images (Canon 10D) under different illuminants and settings. Median dE*ab is 6.40 for the proper patches of calibrated normal images, 17.75 for uncalibrated images.

The accuracy and reproducibility of the colour calibration using different cameras, camera settings and illumination conditions are presented using a probability distribution of dE_ab errors of all the MBCCC patches (Figure 10). A distinction is made between the full set of images and the 'normal' images, which were acquired with proper camera settings: correct manual or automatic white balance and no exposure bias. Indeed, the full set contains several images that were strongly over- or underexposed, or had a mismatched white balance. These images demonstrated the effectiveness of the calibration method, but are not representative of day-to-day photography. Moreover, the term 'proper patch' was used to indicate patches that were not saturated during acquisition i.e. those patches with pixel values too close to 255 or 0. It was not possible to calculate the pixel value of these saturated patches, and their calibration was unfeasible.

**Figure 11:**
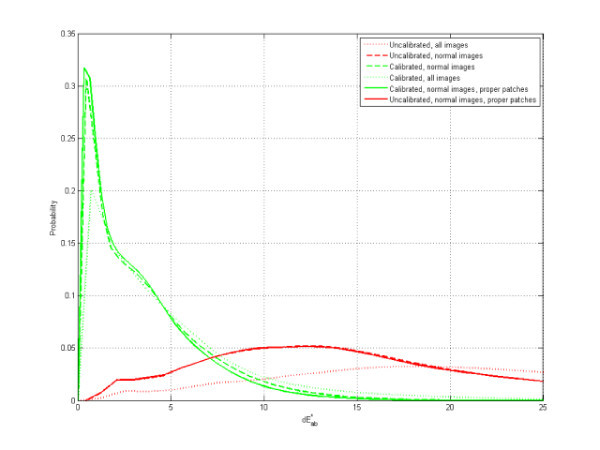
**Reproducibility of Color Calibration**. Probability distribution of dE*ab errors, based on 39 images taken with a Nikon D200 and 15 images with a Canon 10D under different illuminants and settings. Median of 3.43 dE*ab for all calibrated images, 23.26 dE*ab for all uncalibrated images, a median of 2.83 dE*ab for all 'normal' calibrated images and 14.25 dE*ab for all 'normal' uncalibrated images. If we restrict ourselves to the proper patches of normal calibrated images the median is only 2.58 dE*ab

The accuracy and reproducibility results for the set of proper patches of normal images are representative for colours in properly photographed images, which are different from the colours of the patches that were disregarded during calibration due to saturation (marked by 'x' on the calibrated image) (Figure 11). Saturation in normal images or skin imaging is rare, but if it does occur it normally manifests itself as an overexposure of white, deep red, yellow and orange MBCCC patches. If this problem is frequent with a particular camera, it can be remedied by slightly underexposing images by, for example, half an f-stop (exposure bias).

**Figure 12:**
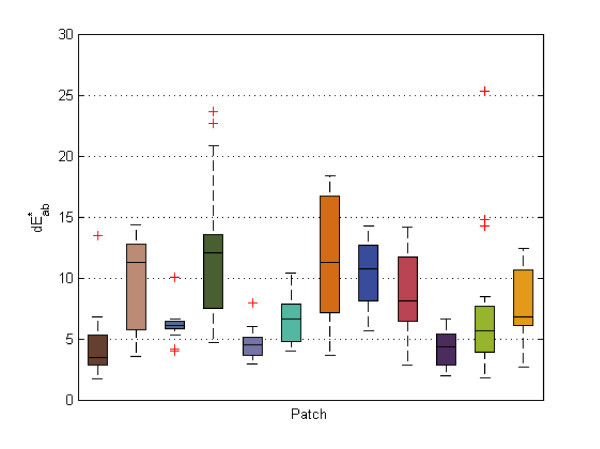
**Accuracy: boxplot for the proper patches of the normal images**.

Tukey's five-number summary of the dE_ab colour differences of each proper patch of the normal images was calculated and visualized using a box plot (the minimum, the lower quartile, the median, the upper quartile and the maximum) (Figure 12). Outliers were marked with a red 'x'. To evaluate accuracy, the chart patches were split in two groups of 12 patches and only the second group was used for calibration, resulting in a lower quality calibration than if 24 patches had been used. The first group of 12 patches was used to check the accuracy.

Colour differences between the measurements and real spectrophotometric measurements revealed median dE_ab values of 6.40 for proper patches of calibrated normal images and 17.75 for uncalibrated images, respectively, demonstrating an important improvement in accuracy after calibration (Figure 10). The result for the patches used in the calibration was also included, and they had a median of 1.59 dE_ab.

Figure 12 presents the accuracy box plot for the proper patches of the normal images. As mentioned above, we could only use patches that had not been used in computing the calibration in order to check accuracy, therefore only 12 patches are shown in this figure.

As figure 11 demonstrates, the reproducibility, visualized by the probability distribution of the dE_ab errors between two measurements of the patches of the images, had a median of 3.43 dE* for all calibrated images, 23.26 dE_ab for all uncalibrated images, a median of 2.83 dE_ab for all 'normal' calibrated images, and 14.25 dE_ab for all 'normal' uncalibrated images. Restricting the calculation to the proper patches of normal calibrated images, the median was 2.58 dE_ab. Wilcoxon sum-rank testing (p < 0.05) between uncalibrated normal images and calibrated normal images with proper squares was equal to zero, demonstrating a highly significant improvement in reproducibility.

**Figure 13:**
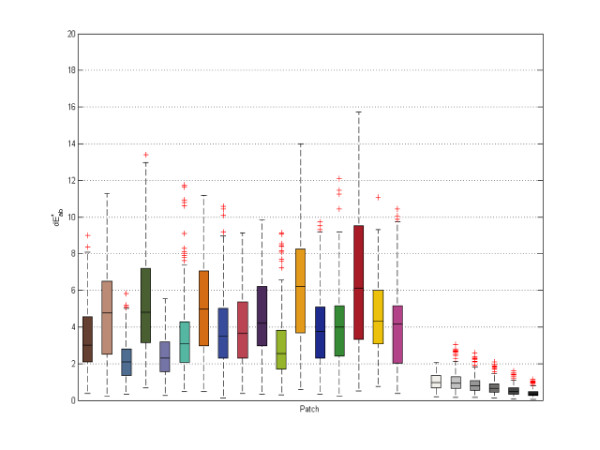
**Reproducibility: boxplot for the proper patches of the normal images**.

Examining dE_ab errors for each MBCCC patch individually revealed that the greatest errors were found in the red, orange yellow, orange and yellow patches. Examination of cyan patches was excluded as these cannot be represented accurately in the sRGB colour space (Figure 13).

### Experiment 2

**Figure 14:**
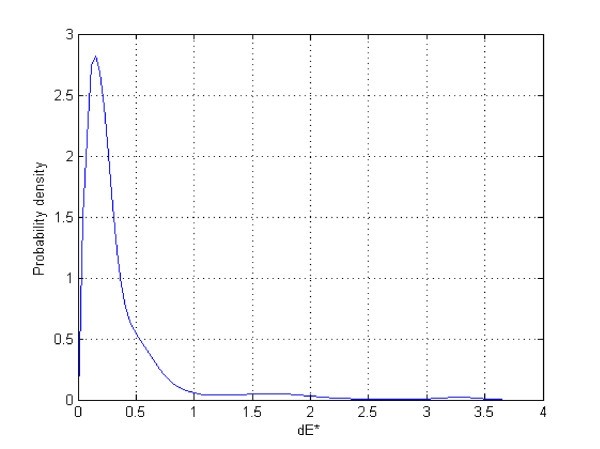
Probability distribution of dE*ab errors with region of interest calibration.

The reproducibility of the chart detection during automatic calibration is presented using a probability distribution of dE_ab errors between two measurements of the same ROI. Ideally this should be as close to zero as possible and comparable to the measurements of the same ROI depicted in the presented figures. Reproducibility: box plot for the proper patches of the normal images. The rotated versions of an image should all be equal. Any deviation from this would indicate variability in the chart detection, leading to a slightly different calibration and thus different measurements (Figure 14).

## Discussion

The research presented here provides evidence that images taken with commercially available digital cameras can be calibrated independently of camera settings and illumination features, provided that illumination in the field of view is uniform and a calibration chart is used. This may be particularly useful during chronic wound assessment, as this is often performed in different locations and under variable lighting conditions. The proposed calibration transforms the acquired images in an unknown colour space (usually RGB) to a standard, well defined colour space (sRGB) that allows images to be displayed properly and has a known relationship to the CIE colorimetric colour spaces. First, we challenged the calibration procedure with a large collection of images containing both 'normal' images with proper camera settings and images that were purposely over- or underexposed and/or had white balance mismatches. The reproducibility and accuracy of the calibration procedure is presented and demonstrates marked improvements. The calibration procedure works very well on the images with improper camera settings, as evidenced by the minimal differences between the error distributions of the complete set of images and the set with only the 'normal' images. An innovative feature demonstrated during our research is the automatic 'detection and calibration of the MacBeth Colour Checker Chart Mini [MBCCC]' in the digital image. Secondly, we tested the effect of this MBCCC chart detection on subsequent real-world colour measurements. Figure 14 demonstrates the probability distribution of errors between two colour measurements of the same region of interest that can be attributed to variations in the chart detection process. The majority of these errors were below 1 dE_ab, demonstrating that the chart detection is robust.

This experiment is part of the research presented by the Woundontology Consortium, which is a semi-open, international, virtual community of practice devoted to advancing the field of research in non-invasive wound assessment by image analysis, ontology and semantic interpretation and knowledge extraction http://www.woundontology.com. The interests of this consortium are related to the establishment of a community driven, semantic content analysis platform for digital wound imaging with special focus on wound bed surface area and color measurements in clinical settings. Current research by the Woundontology Consortium is related to our concerns of the interpretation of clinical wound images without any calibration or reference procedure. Therefore we are investigating techniques to promote standardization. The platform used by this Consortium is based on Wiki technology, a collaborative environment to develop a "woundontology" using the Collaborative Ontology Development Service (CODS) and an image server. Research on wound bed texture analysis is performed by a computer program: "MaZda". This application has been under development since 1998, to satisfy the needs of the participants of the COST B11 European project "Quantitative Analysis of Magnetic Resonance Image Texture" (1998-2002). Additionally, wound bed texture parameter data-mining is analyzed using "RapidMiner" which is one of the world-wide leading open-source data mining solution.

Recently, results on: THE RED-YELLOW-BLACK (R-Y-B) SYSTEM: A COLORIMETRIC ANALYSIS OF CONVEX HULLS IN THE CIELAB COLOR SPACE were presented at the EWMA 2009 conference in Helsinki, Finland.

## Conclusions

To our knowledge, the proposed technology is the first demonstration of a fundamental, and in our opinion, essential tool for enabling intra-individual (in different phases of wound healing) and inter-individual (for features and properties) comparisons of digital images in human wound healing. By implementing this step in the assessment, we believe that scientific standards for research in this domain will be improved [37].

## Competing interests

The authors declare that they have no competing interests.

## Authors' contributions

SVP designed the method and drafted the manuscript. SVP and YV generated the data gave recommendations for their evaluation. PJ, TM, KV provided feedback and directions on the results. All authors read and approved the final manuscript.

## Pre-publication history

The pre-publication history for this paper can be accessed here:

http://www.biomedcentral.com/1471-2342/10/7/prepub

## References

[B1] Haeghen Y Vander Naeyaert JM Consistent cutaneous imaging with commercial digital cameras Arch Dermatol 2006 142 1 42 46 10.1001/archderm.142.1.42 16415385

[B2] Van Geel N Haeghen Y Vander Ongenae K Naeyaert JM A new digital image analysis system useful for surface assessment of vitiligo lesions in transplantation studies Eur J Dermatol 2004 14 3 150 155 15246939

[B3] Kanthraj GR Classification and design of teledermatology practice: What dermatoses? Which technology to apply? 2009 23 8 Journal of the European Academy of Dermatology and Venereology 865 875 10.1111/j.1468-3083.2009.03136.x 19250331

[B4] Aspres N Egerton IB Lim AC Shumack SP Imaging the skin Australas J Dermatol 2003 44 1 19 27 10.1046/j.1440-0960.2003.00632.x 12581077

[B5] Bhatia AC The clinical image: archiving clinical processes and an entire specialty Arch Dermatol 2006 142 1 96 98 10.1001/archderm.142.1.96 16415393

[B6] Hess CT The art of skin and wound care documentation Advances in Skin & Wound Care 2005 18 43 53 10.1097/00129334-200501000-00017 15714037

[B7] Bon FX Briand E Guichard S Couturaud B Revol M J Servant JM Dubertret L Quantitative and kinetic evolution of wound healing through image analysis IEEE Trans Med Imaging 2000 19 7 767 772 10.1109/42.875206 11055792

[B8] Jury CS Lucke TW The clinical photography of herbert brown: a perspective on early 20^th ^century dermatology Clin Exp Dermatol 2001 26 5 449 454 10.1046/j.1365-2230.2001.00856.x 11488837

[B9] Phillips K Incorporating digital photography into your wound-care practice Wound Care Canada 2006 16 18

[B10] Oduncu H Hoppe A Clark M Williams RJ Harding KG Analysis of skin wound images using digital colour image processing: a preliminary communication Int J Low Extrem Wounds 2004 3 3 151 156 10.1177/1534734604268842 15866806

[B11] Levy JL Trelles MA Levy A Besson R Photography in dermatology: comparison between slides and digital imaging J Cosmet Dermatol 2003 2 131 134 10.1111/j.1473-2130.2004.00081.x 17163918

[B12] Tucker WFG Lewis FM Digital imaging: a diagnostic screening tool? Int J Dermatol 2005 44 6 479 481 10.1111/j.1365-4632.2005.01990.x 15941435

[B13] Wagner JH Miskelly GM Background correction in forensic photography. ii. Photography of blood under conditions of non-uniform illumination or variable substrate color_practical aspects and limitations J Forensic Sci 2003 48 3 604 613 12762531

[B14] Wagner JH Miskelly GM Background correction in forensic photography. i. photography of blood under conditions of non-uniform illumination or variable substrate color_theoretical aspects and proof of concept J Forensic Sci 2003 48 3 593 603 12762530

[B15] Riley RS Ben-Ezra JM Massey D Slyter RL Romagnoli G Digital photography: A primer for pathologists J Clin Lab Anal 2004 18 91 128 10.1002/jcla.20009 15065212 PMC6807831

[B16] Palioto DB Sato S Ritman G Mota LF Caffesse RG Computer assisted image analysis methods for evaluation of periodontal wound healing Braz Dent J 2001 12 167 172 11696912

[B17] Heydecke G Schnitzer S Türp JC The colour of human gingiva and mucosa: visual measurement and description of distribution Clin Oral Invest 2005 9 257 265 10.1007/s00784-005-0006-3 16177882

[B18] Scheinfeld N Photographic images, digital imaging, dermatology, and the law Arch Dermatol 2004 140 4 473 476 10.1001/archderm.140.4.473 15096377

[B19] Gopalakrishnan D Colour analysis of the human airway wall 2003 Master's thesis, University of IOWA

[B20] Lotto RB Purves D The empirical basis of colour perception Consciousness and Cognition 2002 11 609 629 10.1016/S1053-8100(02)00014-4 12470626

[B21] Prasad S Roy B Digital photography in medicine J Postgrad Med 2003 49 4 332 336 14699233

[B22] Maglogiannis I Kosmopoulos DI A system for the acquisition of reproducible digital skin lesions images Technol Health Care 2003 11 6 425 441 14757921

[B23] Haeghen Y Vander Development of a dermatological workstation with calibrated acquisition and management of colour images for the follow-up of patients with an increased risk of skin cancer 2001 Ph.D. thesis, University Ghent

[B24] Gilmore S Modelling skin disease: lessons from the worlds of mathematics, physics and computer science Australas J Dermatol 2005 46 2 61 69 10.1111/j.1440-0960.2005.00143.x 15842395

[B25] Goldberg DJ Digital photography, confidentiality, and teledermatology Arch Dermatol 2004 140 4 477 478 10.1001/archderm.140.4.477 15096378

[B26] Macaire L Postaire JG Colour image segmentation by analysis of subset connectedness and colour homogeneity properties Computer Vision and Image Understanding 2006 102 105 116 10.1016/j.cviu.2005.12.001

[B27] Streinera DL Precision and accuracy: Two terms that are neither Journal of Clinical Epidemiology 2006 59 327 330 10.1016/j.jclinepi.2005.09.005 16549250

[B28] Feit J Ulman V Kempf W Jedlickov H Acquiring images with very high resolution using a composing method Cesk Patol 2004 40 2 78 82 15233022

[B29] Byrne A Hilbert DR Colour realism and colour science Behavioral and Brain Sciences 2006 26 3 64 10.1017/s0140525x03000013 14598439

[B30] Harkness N The colour wheels of art, perception, science and physiology Optics and Laser Technology 2006 38 219 229 10.1016/j.optlastec.2005.06.010

[B31] Multimedia systems and equipment - Colour measurement and management -Part 2-1: Colour management - Default RGB colour space - sRGB 1999 IEC 61966-2-1 Ed. 1.0 Bilingual

[B32] Haeghen Y Vander Naeyaert JM Lemahieu I Philips W An imaging system with calibrated colour image acquisition for use in dermatology IEEE Trans Med Imaging 2000 19 7 722 730 10.1109/42.875195 11055787

[B33] Ikeda I Urushihara K Ono T A pitfall in clinical photography: the appearance of skin lesions depends upon the illumination device Arch Dermatol Res 2003 294 438 443 12563541 10.1007/s00403-002-0360-9

[B34] Leon K Mery D Pedrischi F Leon J Colour measurement in l*a*b* units from rgb digital images Food Research International 2006 39 2006 1084 1091 10.1016/j.foodres.2006.03.006

[B35] Danilova MV Mollon JD The comparison of spatially separated colours Vision Res 2006 46 6-7 823 836 10.1016/j.visres.2005.09.026 16288793

[B36] Johnson GM A top down description of s-cielab and ciede2000 Col Res Appl 2003 28 425 435 10.1002/col.10195

[B37] Bellomo R Bagshaw SM Evidence-based medicine: classifying the evidence from clinical trials_the need to consider other dimensions Crit Care 2006 10 5 232 10.1186/cc5045 17029653 PMC1751050

